# Comparison of the protective effect of human respiratory syncytial virus Pre-F protein combined with different adjuvants in BALB/c mice

**DOI:** 10.3389/fimmu.2026.1904272

**Published:** 2026-07-20

**Authors:** Mengxuan Chu, Liang Du, Hongqiao Hu, Lei Cao, Li Zhang, Naiying Mao, Zhen Zhu, Yan Zhang, Hai Li

**Affiliations:** 1 National Key Laboratory of Intelligent Tracking and Forecasting for Infectious Disease, National Institute for Viral Disease Control and Prevention, Chinese Center for Disease Control and Prevention (Chinese Academy of Preventive Medicine), Beijing, China; 2 National Health Commission (NHC) Key Laboratory of Medical Virology and Viral Diseases, National Institute for Viral Disease Control and Prevention, Chinese Center for Disease Control and Prevention (Chinese Academy of Preventive Medicine), Beijing, China; 3 Shandong First Medical University & Shandong Academy of Medical Sciences, School of Public Health and Health Management, Jinan, Shandong, China

**Keywords:** adjuvant, human respiratory syncytial virus, neutralizing antibody, pre-F protein, vaccine

## Abstract

**Introduction:**

Human respiratory syncytial virus (HRSV) has a high disease burden in infants and elderly individuals. In this study, the adjuvants AlOH, AlOH+CpG and BFA03 were used to compare the protective effect of HRSV prefusion protein (Pre-F) in BALB/c mice.

**Methods:**

We divided BALB/c mice into three experimental groups (Pre-F+AlOH+CpG, Pre-F+AlOH, and Pre-F+BFA03) and three adjuvant control groups (AlOH+CpG, AlOH, and BFA03). After two intramuscular immunizations, we measured serum neutralizing antibody titers and quantified the numbers of IFN-γ- and IL-4-secreting lymphocytes. After viral challenge, we monitored body weight changes, determined lung viral loads (Ct values), and scored lung pathological damage.

**Results:**

The mice in the experimental groups exhibited high titres of neutralizing antibodies and increased numbers of IFN-γ- and IL-4-secreting lymphocytes. The mice began to regain weight on the third day after challenge, but the mice in the adjuvant groups continued to lose weight. The mice immunized with Pre-F+BFA03 elicited the highest neutralizing antibody titre (1716), the lowest viral load in the lung, and milder pathological damage. The mice immunized with Pre-F+AlOH had the most severe lung pathological injury (score: 2.83) and the highest viral load in the lung (Ct value: 32.2). Compared with the BFA03 adjuvant, the AlOH adjuvant induced a Th2-biased humoral immune response in mice. The Pre-F+AlOH+CpG group had the least pathological damage in the lung (score 2.16), and the ability to induce neutralizing antibodies and cellular immune responses was comparable with that of the BFA03 adjuvant.

**Discussion:**

These findings indicate that Pre-F protein combined with AlOH+CpG or BFA03 adjuvant provided similar protection in mice and both were superior to the AlOH adjuvant, providing a reference for adjuvant selection and formulation strategies in HRSV Pre-F protein vaccine development.

## Introduction

Human respiratory syncytial virus (HRSV) is the only virus in the genus *Orthopneumovirus* of the family Pneumoviridea to infect humans, and it is also known as human Orthopneumovirus (1). HRSV contains 10 genes, which can encode 11 proteins, including two nonstructural proteins (NS1 and NS2), nucleocapsid protein (N), phosphoprotein (P), matrix protein (M), small hydrophobic protein (SH), glycoprotein (G), fusion protein (F), and M2–1 and M2–2 proteins, which can be expressed by the M2 gene and RNA-dependent RNA polymerase (L) (2). A variety of HRSV vaccines have been designed based on the structural proteins F, G, and SH. The F protein includes two structures, prefusion protein (Pre-F) and postfusion protein (Post-F), which can mediate the fusion of the viral envelope and the host cell membrane through conformational transition, and is highly conserved. The Pre-F protein conformation includes six epitopes (site I, II, III, IV, V, and Ø) and only four epitopes (site I, II, III, and IV) for Post-F, where site III is predominantly present in Pre-F and site I is predominantly present in Post-F (3). Site Ø is located at the top of the distal membrane of the Pre-F protein, and the recognition sites are aa 62–69 and aa 196-209. The neutralizing activity of antibodies induced by site Ø is much higher than that of other epitopes. Therefore, the stable site Ø is an ideal neutralizing epitope for HRSV vaccine development, and vaccine development is expected to contain a stable site Ø (4).

HRSV is mainly transmitted by droplets into and adsorbed on the respiratory mucosa, causing upper respiratory tract infection, and in severe cases it can cause lower respiratory tract infection. It mainly infects infants, elderly individuals, and people with low immunity, and it is the most common viral pathogen of acute lower respiratory tract infection in infants and young children (5). Almost all infants and young children under 2 years old have been infected with HRSV, which often causes lower respiratory tract infection, usually manifested as bronchiolitis and pneumonia, while neonates younger than 3 months have more severe symptoms (6). One study estimated that in 2019 more than 45, 000 infants younger than 6 months died from acute lower respiratory infections caused by HRSV (7). Severe HRSV bronchiolitis in infancy and early childhood is a strong risk factor for the development of allergic asthma in early adolescence (8). Children and adults usually have upper respiratory tract infections, and the symptoms are usually mild (9). With increasing age, the underlying diseases of elderly individuals increase, and immunity decreases. Therefore, HRSV also has high morbidity and mortality in the elderly (10). In addition, HRSV infection can cause extrapulmonary organ diseases, including interstitial myocarditis, epilepsy, or encephalitis (11).

The immune population of the HRSV vaccine includes infants, pregnant women, and elderly individuals. The specific antibody levels in these three groups are different, and the immune system maturity is different; thus, it is difficult to develop a vaccine that is suitable for all three groups at the same time. A large number of studies have shown that nonreplicating vaccines should be avoided in infants who have not been infected with HRSV (12). The elderly have been naturally infected with HRSV many times, and it is difficult for the live-attenuated vaccine to induce the ideal protective effect, and the stimulated immune response is not sufficient to prevent HRSV reinfection (13). Therefore, the HRSV vaccines used in clinical research need to have the appropriate vaccine form or adjuvant according to the different target populations to solve the problem of HRSV infection faced by different populations. At present, the vast majority of vaccines and monoclonal antibodies were designed based on the Pre-F protein. Three HRSV vaccines have been approved globally: preF3 (Arexvy) (14), preF (Abrysvo) (15), and mRNA-1345 (16). These vaccines are indicated for adults aged 60 years and older, as well as adults aged 18–59 years who are at increased risk of RSV-associated lower respiratory tract disease (17). Although no HRSV vaccine has yet been approved for infants, three RSV monoclonal antibodies for infant use have been approved globally: nirsevimab, clesrovimab, and palivizumab (18, 19).

Formalin-inactivated RSV vaccines (FI-RSV) induce the body to produce a biased Th2 immune response, causing enhanced respiratory disease (ERD) (20); therefore, ideal HRSV vaccines need to induce the body to produce a biased Th1 immune response. Adjuvants can not only enhance the strength of the body’s immune response but also regulate the bias of the immune response. Aluminium adjuvants, including aluminium phosphate (Al-Phos) and aluminium hydroxide (AlOH), are the most commonly used vaccine adjuvants for human use. The antigen adsorbed by aluminium adjuvant is slowly released at the injection site and provides long-term and effective stimulation of the immune system; however, it can cause granulomas at the injection site, attract cells of the innate immune system, such as monocytes, eosinophils, and neutrophils (21), and induce the body to produce a Th2-biased immune response (22). CpG oligodeoxynucleotides (CpG), a commonly used adjuvant, are Toll-like receptor 9 (TLR9) agonists. TLR9 is mainly expressed in dendritic cells (DCs) and B cells in humans and mice and is a strong inducer of B-cell activation, plasmacytoid dendritic cell maturation, and monocyte maturation. CpG can enhance the immune activity of vaccine-induced cytotoxic T cells and induce the production of Th1-biased immune responses (23), which is helpful for solving the problems of insufficient safety and weak immunogenicity of HRSV vaccines. CpG has been successfully used as an adjuvant in the hepatitis B vaccine. The combination of CpG ODN and aluminium adjuvant has a synergistic effect to promote the balance of the Th1/Th2 immune response.

The use of novel adjuvants is the secret for recombinant protein vaccines to excel. At present, a variety of novel adjuvants have been applied to viral vaccines, including AS01B adjuvant for herpes zoster vaccine, AS04 adjuvant for human papillomavirus (HPV) vaccine, Matrix M1 adjuvant for COVID-19 vaccine, and MF59 adjuvant for influenza virus vaccine. Experts of FDA agreed that the RSV PreF3 vaccine (developed by GSK) is highly effective, and it benefits from the strong humoral and cellular immune responses induced by the use of the AS01E adjuvant. BFA03 (α-tocopherol, squalene, and polysorbate 80), developed by Reco Bio, has been tested in clinical trials for COVID-19 recombinant protein vaccines. The AS01E adjuvant was not commercially available as a standalone reagent at the time of this study; therefore, we focused on comparing AlOH (a widely used aluminum adjuvant), AlOH+CpG (a TLR9 agonist combination), and BFA03 (a novel candidate adjuvant). Future studies directly comparing these formulations with AS01E would be of significant value.

In this study, BALB/c mice were immunized by intramuscular injection with AlOH, AlOH+CpG or BFA03 adjuvant mixed with Pre-F (CHO cell expression). By comparing the immunogenicity of the Pre-F protein in BALB/c mice, the effects of different adjuvants on the immunogenicity of the Pre-F protein were evaluated. This study provides a theoretical basis for further research on adjuvanted HRSV vaccines.

## Materials and methods

### Viruses, BALB/c mice and Pre-F protein

HEp-2 cells and the HRSV-Long strain were stored in our laboratory. Female BALB/c mice aged 6–8 weeks were purchased from Vitong Lihua Laboratory Animal Technology Co., Ltd. in Beijing. The animal experiments involved in this study were approved by the Animal Experiment Ethics Committee of the National Institute for Virus Disease Control and Prevention, Chinese Center for Disease Control and Prevention.

Pre-F protein was expressed in CHO-S cells (purchased from Invitrogen). Pre-F contains six amino acid mutations (S155C, S290C, A149C, Y458C, S190F, and V207C), and a foldon sequence (GYIPEAPRDGQAYVRKDGEWVLLSTFL) and His tag were added at the C-terminus. After affinity chromatography, the purity and structure of Pre-F were verified by SDS–PAGE and transmission electron microscopy.

### Immunization strategies

Thirty-six female BALB/c mice (6–8 weeks old) were randomly divided into 6 groups (6 mice in each group). The mice were immunized intramuscularly with 5 μg of Pre-F and adjuvant on Days 0 and 21 (**Table 1**). The mice were challenged 14 days after the second immunization (2×10^5^ PFU/mouse). This challenge dose was selected based on our preliminary dose-finding experiments, which demonstrated that this dose induces detectable viral replication, significant body weight loss, and histopathological changes in BALB/c mice without causing excessive mortality, thereby allowing clear differentiation of protective efficacy among vaccine groups. The same dose was used for all groups to ensure comparability of the results. The changes in body weight were observed and recorded daily. The spleen and lungs of mice were removed aseptically for testing on the fifth day after challenge.

**Table 1:** Animal grouping and immunizations.

Group	Antigen	Adjuvant and dose	Immunization	Number
Pre-F+AlOH+CpG	Pre-F	25 μg CpG+50 μg AlOH	0, 21	6
Pre-F+AlOH	Pre-F	50 μg AlOH	0, 21	6
Pre-F+BFA03	Pre-F	50 μL BFA03	0, 21	6
AlOH+CpG	–	25 μg CpG+50 μg AlOH	0, 21	6
AlOH	–	50 μg AlOH	0, 21	6
BFA03	–	50 μL BFA03	0, 21	6

### IgG, IgG1 and IgG2a antibodies were detected by ELISA

Specific IgG, IgG1, and IgG2a antibodies in mouse serum were detected by ELISA. The procedure was as follows: each well of the 96-well microplate was coated with 20 ng of Pre-F protein and incubated at 4 °C overnight. The next day, the microplate was blocked with PBS containing 3% BSA for 2 hours, washed, dried, and used for subsequent 4-fold dilution of the collected mouse serum (from 1:100). After incubation at 37 °C for 1 h, horseradish peroxidase (HRP)-labelled goat anti-mouse IgG (1:5000 dilution) and HRP-labelled goat anti-mouse IgG1 and IgG2a (1:50000 dilution) were added. After incubation at 37 °C for 1 h, TMB colour was observed. The cells were incubated at 37 °C for 15 min, and the absorbance (OD) was measured by a microplate reader (wavelength 450 nm) after the termination solution was added. EC50 values were calculated by 4-parameter fitting using GraphPad Prism software.

### Neutralizing antibody titre was detected by plaque reduction neutralization test

HEp-2 cells were seeded onto 24-well plates one day in advance. A twofold ratio dilution (starting at 1:32) was performed on 60 μL of serum. The same volume of 50 PFU virus suspension was added to the diluted serum, and the mixture was mixed and neutralized in a 37 °C, 5% CO_2_ incubator for 2 hours. Then, the mixture was added to the cell wells, and each dilution was 2 wells. The cells were adsorbed for 1 hour. Inoculants were removed, and the overlay was added and cultured at 37 °C in a 5% CO_2_ incubator for 5 days. On Day 5, the overlay was removed by aspiration, the cells were fixed and stained with 0.5% crystal violet, and the number of plaques was counted. Neutralizing antibody titres were calculated using the Reed–Muench formula as the reciprocal of the serum dilution for which the number of plaques was reduced by 50%.

### The number of lymphocytes secreting IFN-γ and IL-4 was detected by ELISPOT

Mouse spleen tissue was aseptically removed and placed into a culture dish containing 4 mL of mouse lymphocyte separation solution, and the spleen was ground with the inner core of a syringe. After grinding, the solution was transferred to a 15 mL centrifuge tube and covered with 1 mL of RPMI 1640 medium. After centrifugation at 800 × g for 25 min, the lymphocyte layer was aspirated, and 10 mL of RPMI 1640 medium was added, mixed, and centrifuged at 250 g for 5 min. Mouse spleen cells were collected and diluted to 1x10^6^ cells/mL by adding culture medium. Each well was stimulated with 10 μL of Pre-F protein (10 μg/mL), the positive control was stimulated with 10 μL of positive stimulator (PMA+ionomycin), and the negative control was stimulated with 10 μL of culture medium. ELISPOT was used to detect the number of IFN-γ- and IL-4-secreting lymphocytes. Other procedures were performed according to the instructions of the ELISPOT kit (Daktronics Biotech, batch number: 2210002, 2210402).

### Cycle threshold (Ct) of HRSV nucleic acid of lung tissue

The right lung tissue of mice was removed aseptically, and 2% DMEM was added for grinding. Two hundred microlitres was taken to extract nucleic acid, and the pulmonary viral load of HRSV in lung tissue was detected by real-time quantitative fluorescent PCR (RT–PCR).

### Pathological injury of lung tissue

The left lung tissue was fixed with 4% paraformaldehyde for 24 hours and stained with haematoxylin and eosin (HE) to examine the characteristics of tissue sections, including alveolitis, bronchiolitis, and infiltration of inflammatory cells around blood vessels and in the tissue space. The lung tissues of individual mice were blindly evaluated by a pathologist at Beijing Lanrui Pu Technology Co., Ltd. The score was 0 (normal, no lesion), 1 (mild lesion, where the lesion area was less than 1/4 of the lung tissue section), 2 (moderate lesion, where the lesion area was approximately 1/4 to 2/4 of the lung tissue section), 3 (severe lesion, where the lesion area was approximately 2/4 to 3/4 of the lung tissue section), and 4 (very severe lesion, where the lesion area was more than 3/4 of the lung tissue section).

### Statistical analysis

Excel software was used for data collation, and GraphPad Prism 9.0 software was used for statistical analysis and drawing. One-way analysis of variance was used to calculate whether a significant difference existed between groups. If *P* < 0.05, the difference was statistically significant and marked with *.

## Results

### Modification and analysis of the Pre-F protein

**Figure 1:**
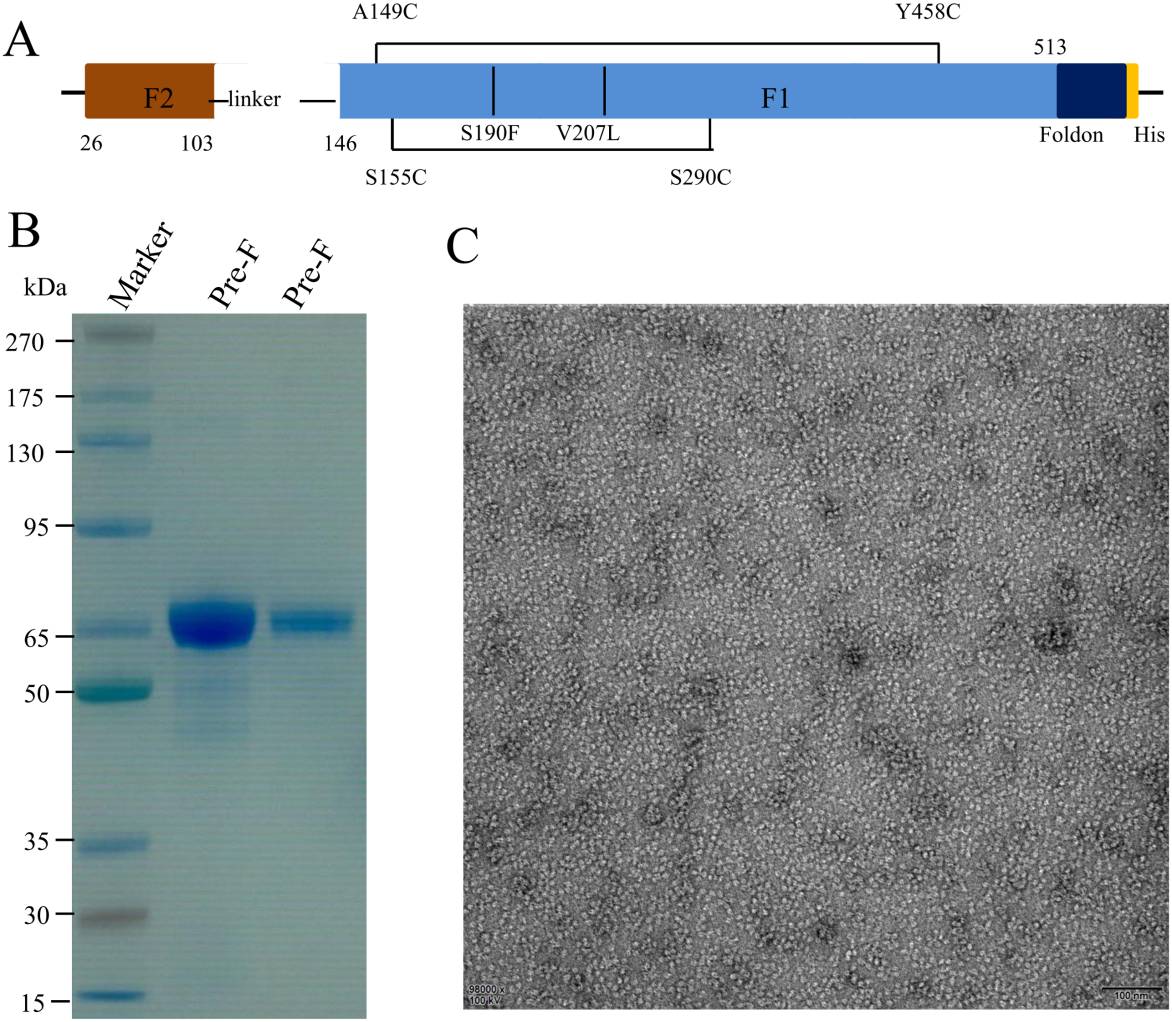
Modification and analysis of the Pre-F protein. **(A)** Schematic diagram of the Pre-F construct. The construct consists of the F2 subunit (brown), linker, and F1 subunit (light blue), followed by a C-terminal T4 fibritin trimerization motif (Foldon, dark blue), a thrombin cleavage site, and a 6× His tag (yellow). **(B)** SDS-PAGE analysis of the purified Pre-F protein. **(C)** Transmission electron microscopy (TEM) observation of the Pre-F protein.

Pre-F protein was expressed in CHO-S cells (purchased from Invitrogen). Pre-F contains six amino acid mutations (S155C, S290C, A149C, Y458C, S190F, and V207C), and a foldon sequence (GYIPEAPRDGQAYVRKDGEWVLLSTFL) and His tag were added at the C-terminus (**Figure 1A**). After affinity chromatography, the molecular weight of Pre-F was approximately 65 kDa (**Figure 1B**). The uniform trimer structure can be seen under transmission electron microscopy (**Figure 1C**).

### Humoral immune responses in mice

**Figure 2:**
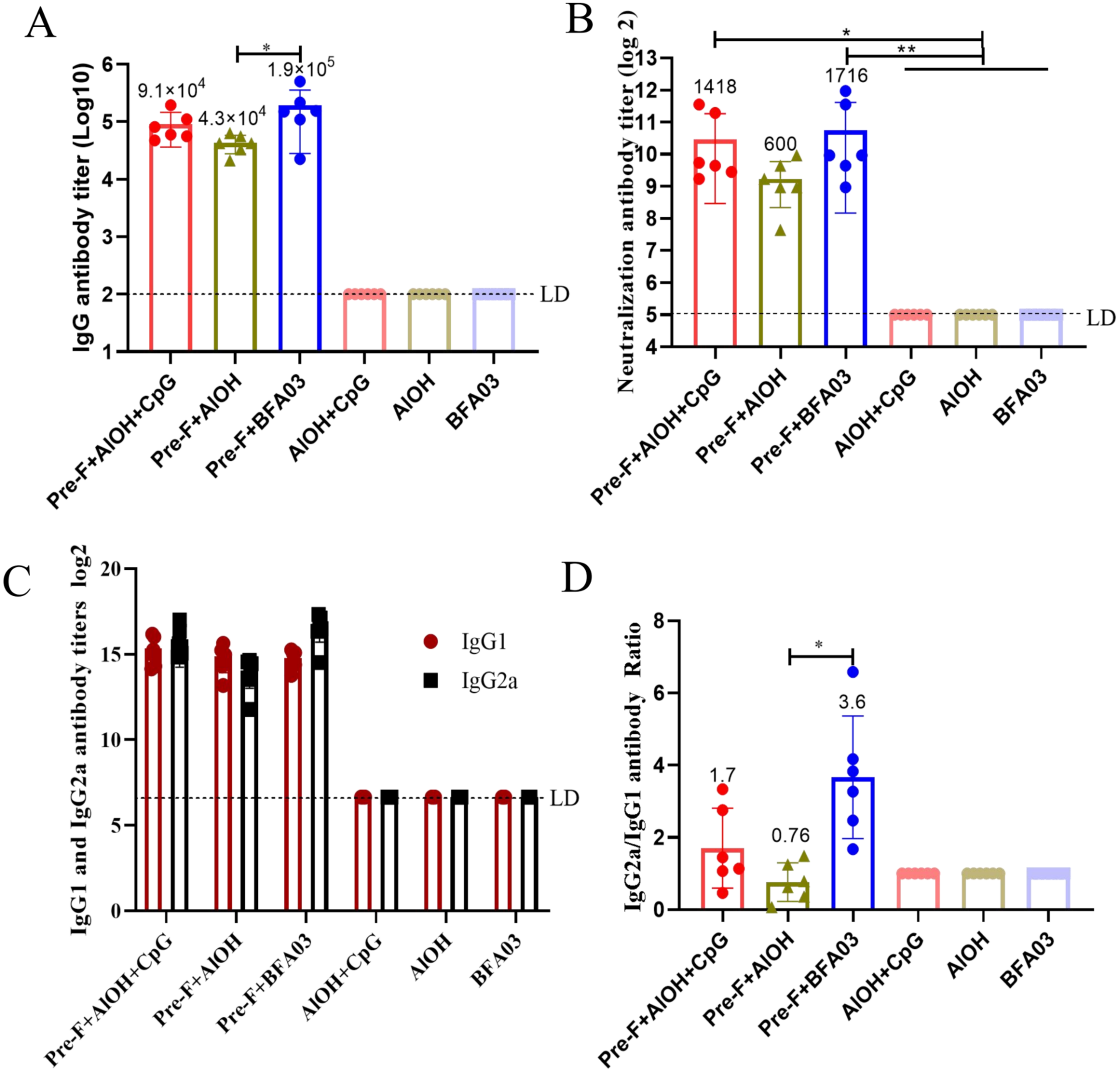
Humoral immune response in mice. **(A)** IgG antibody titres in serum on Day 35. **(B)** Serum neutralizing antibody titres of mice. **(C)** IgG1 and IgG2a titres in serum. **(D)** Ratio of IgG2a to IgG1 antibody. * indicates P< 0.05, ** indicates P< 0.01.

To verify the humoral immune responses induced by the three adjuvants, indirect ELISA was used to detect the specific IgG antibody titres in the serum of mice. As shown in **Figure 2A**, the mice in the experimental groups (Pre-F+AlOH+CpG, Pre-F+AlOH, and Pre-F+BFA03) produced higher IgG antibodies than those in the adjuvant groups (AlOH+CpG, AlOH, and BFA03). The IgG antibody titre of the Pre-F+AlOH immunization group was the lowest (4.3×10^4^) and was significantly lower than that of the Pre-F+BFA03 immunization group (*P* < 0.05). The IgG antibody titre of the Pre-F+BFA03 immunization group was the highest (1.9×10^5^) but not significantly higher than that of the Pre-F+AlOH+CpG immunization group (9.1×10^4^) (*P* = 0.1673).

Neutralizing antibodies are one of the important parameters for evaluating the protective effect of the HRSV vaccine. In this study, a plaque reduction neutralization test was used to evaluate the level of neutralizing antibody induced by three adjuvants in mice. As shown in **Figure 2B**, high titres of neutralizing antibodies were induced in all experimental groups (Pre-F+AlOH+CpG, Pre-F+AlOH, and Pre-F+BFA03), and the differences in neutralizing antibody titres among the three experimental groups were similar to those of IgG antibodies. The Pre-F+BFA03 group had the highest neutralizing antibody titre (1716), and the Pre-F+AlOH group had the lowest neutralizing antibody titre (600). Similarly, although the neutralizing antibody titer in the Pre-F+AlOH+CpG group (1418) was higher than that in the Pre-F+AlOH group (600), this difference did not reach statistical significance (*P* = 0.417).

To further verify the bias of the humoral immune response induced by the three adjuvanted mice, the antibody titres of IgG1 and IgG2a in serum were detected by ELISA. **Figure 2C** shows that the antibody titres of IgG2a in the Pre-F+AlOH+CpG group and Pre-F+BFA03 group were higher than those in the IgG1 group. However, the IgG1 antibody titre of the Pre-F+AlOH immunization group was higher than that of IgG2a. As shown in **Figure 2D**, comparing the ratio of IgG2a and IgG1 antibodies, the ratio of IgG2a/IgG1 in the Pre-F+AlOH immunization group (0.76) was less than 1, which was significantly lower than that in the Pre-F+BFA03 immunization group (*P* < 0.05). At the same time, although it was numerically lower than that of the Pre-F+AlOH+CpG immunization group, this difference did not reach statistical significance (*P* = 0.4216). Compared with the Pre-F+AlOH immunization group, the Pre-F+BFA03 and Pre-F+AlOH+CpG immunization groups induced a more Th1-based immune responses profile in mice.

### Cellular immune responses in mice

**Figure 3:**
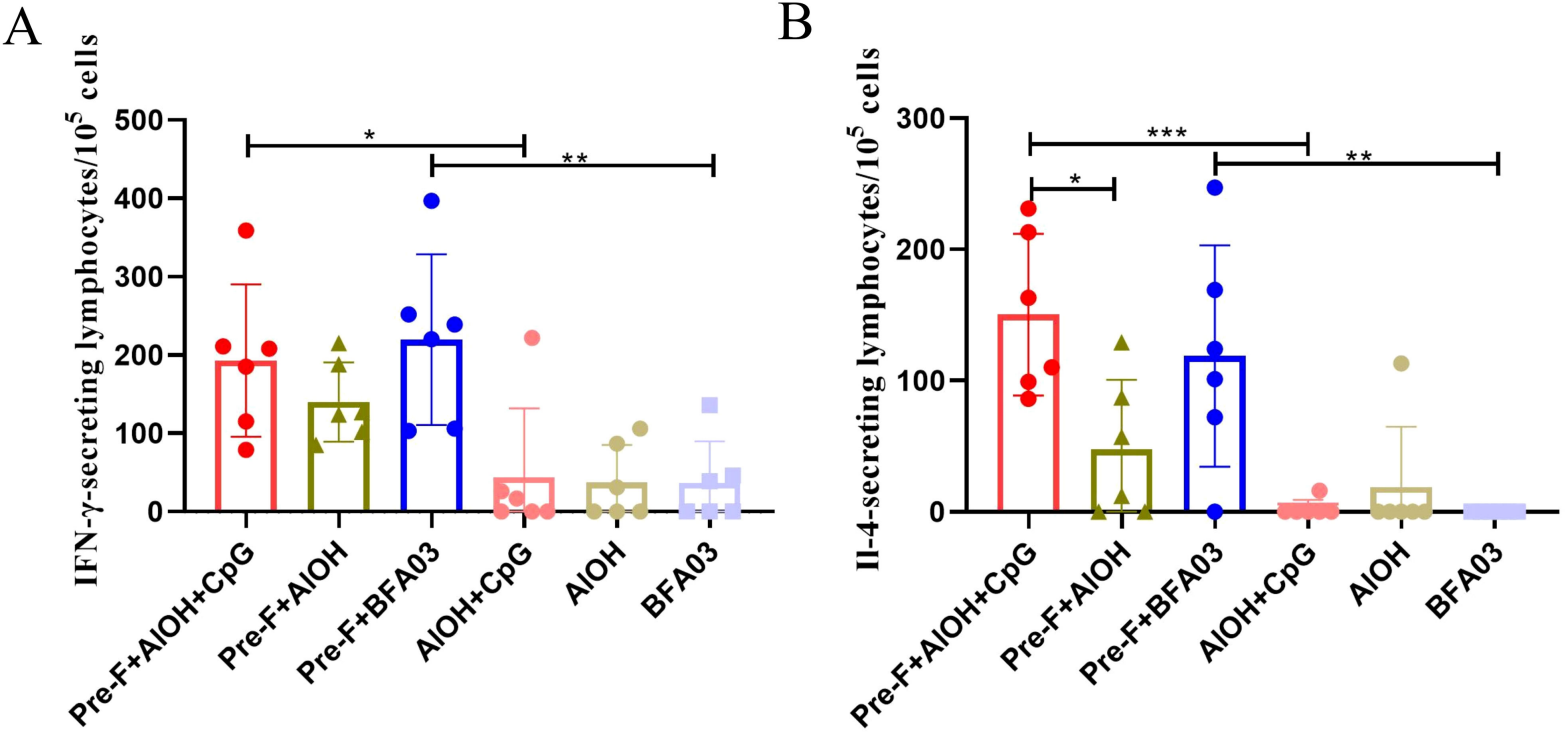
Cellular immune response in mice. **(A)** The number of lymphocytes secreting IFN-γ. **(B)** The number of lymphocytes secreting IL-4. * indicates P< 0.05, ** indicates P< 0.01, and *** indicates P< 0.001.

ELISPOT was used to detect the intensity of cellular immune responses. As shown in **Figure 3A**, the number of IFN-γ-secreting lymphocytes in the Pre-F+BFA03 group was significantly higher than that in the BFA03 adjuvant group (*P* < 0.01). The number of IFN-γ-secreting lymphocytes in the Pre-F+AlOH+CpG immunization group was significantly higher than that in the AlOH+CpG adjuvant group (*P* < 0.05). Similarly, as shown in **Figure 3B**, the number of IL-4-secreting lymphocytes in the Pre-F+BFA03 and Pre-F+AlOH+CpG immunization groups was significantly higher than that in the BFA03 and AlOH+CpG adjuvant groups (*P* < 0.01). The number of IL-4-secreting lymphocytes in the Pre-F+AlOH immunization group was significantly lower than that in the Pre-F+AlOH+CpG immunization group (*P* < 0.05). In the Pre-F+AlOH+CpG immunization group, the number of IFN-γ-secreting lymphocytes was significantly higher than that of IL-4-secreting lymphocytes (*P* < 0.05). Among the three experimental groups, the number of IFN-γ- and IL-4-secreting lymphocytes in the Pre-F+AlOH group was the lowest, indicating that the ability of the AlOH adjuvant to induce a cellular immune response was weak, but the number of IFN-γ- and IL-4-secreting lymphocytes in the Pre-F+ ALOH group was increased after combination with the CpG adjuvant.

### Pathological changes and viral load in the lungs of mice were observed

**Figure 4:**
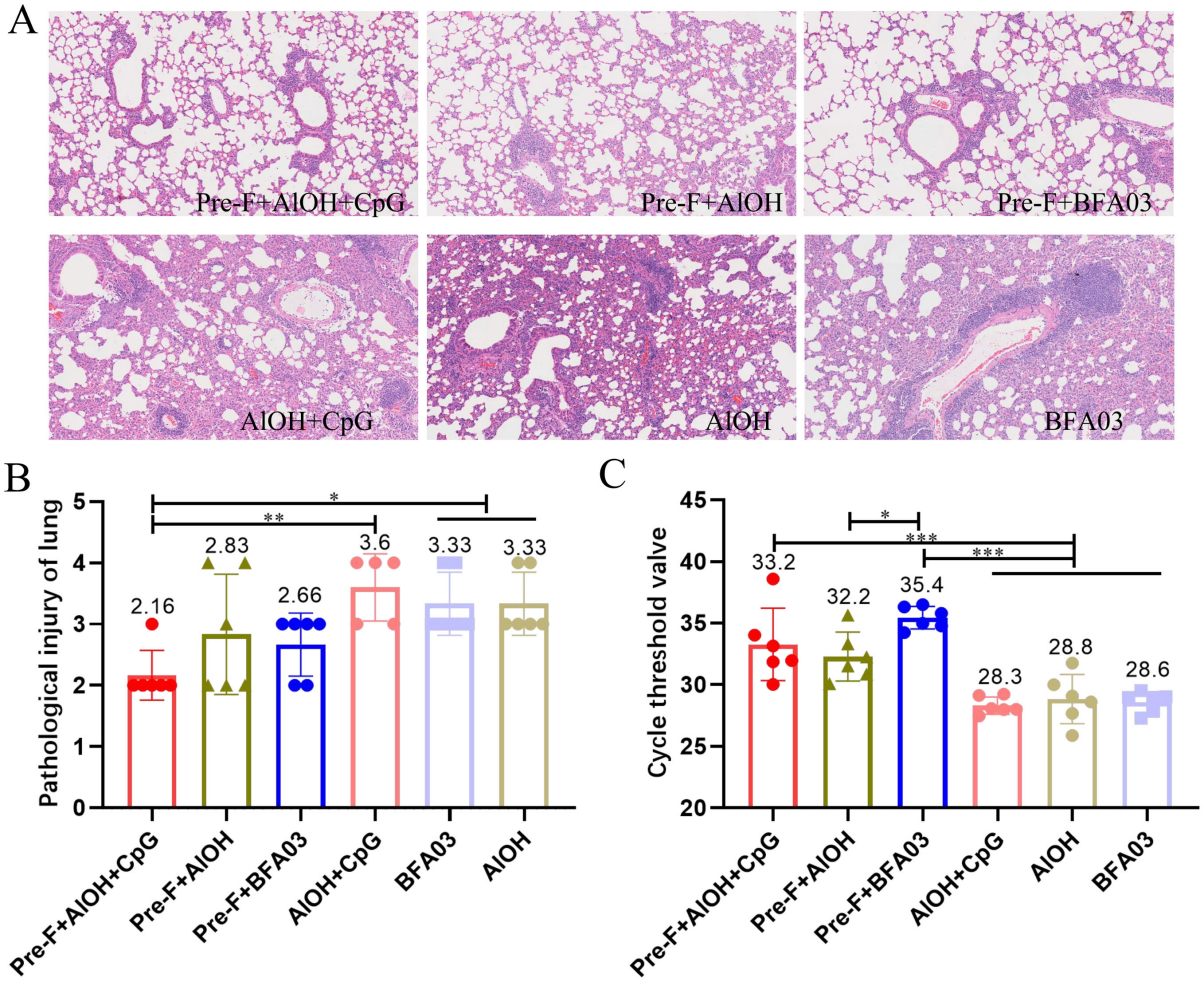
Histopathology analysis of the lungs and HRSV load in the lungs. **(A)** Lungs were stained with haematoxylin and eosin (H&E) for histologic evaluation. **(B)** Comprehensive score of lung pathological injury in mice. **(C)** Ct value of HRSV on Day 5 after virus challenge. * indicates P< 0.05, ** indicates P< 0.01, and *** indicates P< 0.001.

On the fifth day after challenge (2×10^5^ PFU/mouse), the lung tissue was removed aseptically, and the pathological damage to the lung tissue was analysed after HE staining. The pathological scores were scored according to the scoring standard by the pathologists of Beijing Lanrui Pu Technology Co., Ltd. As shown in **Figure 4A**, the mice in the adjuvant groups (AlOH+CpG, AlOH, and BFA03) had thickened alveolar walls and a large number of inflammatory cells infiltrated around the blood vessels or bronchi, while the mice in the experimental groups (Pre-F+AlOH+CpG, Pre-F+AlOH, and Pre-F+BFA03) had less pathological damage. The alveolar walls were thin and delicate, and the extent of the lesion was small. As shown in **Figure 4B**, for the comprehensive judgement score of lung lesions, the pathological scores of the experimental group were lower than those of the adjuvant group, and the score of the Pre-F+AlOH+CpG immunization group was the lowest (2.16), followed by that of the Pre-F+BFA03 immunization group (2.66). The lung pathological injury of mice in the three adjuvant groups was more serious, and the scores were all higher than 3.

The Ct values of HRSV viral nucleic acid in the lung tissue of mice were detected by RT–PCR. As shown in **Figure 4C**, the Ct values of Pre-F+AlOH+CpG and Pre-F+BFA03 in the experimental group were higher than those in the three adjuvant groups (*P* < 0.001). The average Ct value of the Pre-F+BFA03 immunization group was 35.4, and the average Ct value of the Pre-F+AlOH immunization group was 32.2, which was significantly lower than that of the Pre-F+BFA03 immunization group (*P* < 0.05).

### Changes in body weight of the mice after challenge

**Figure 5:**
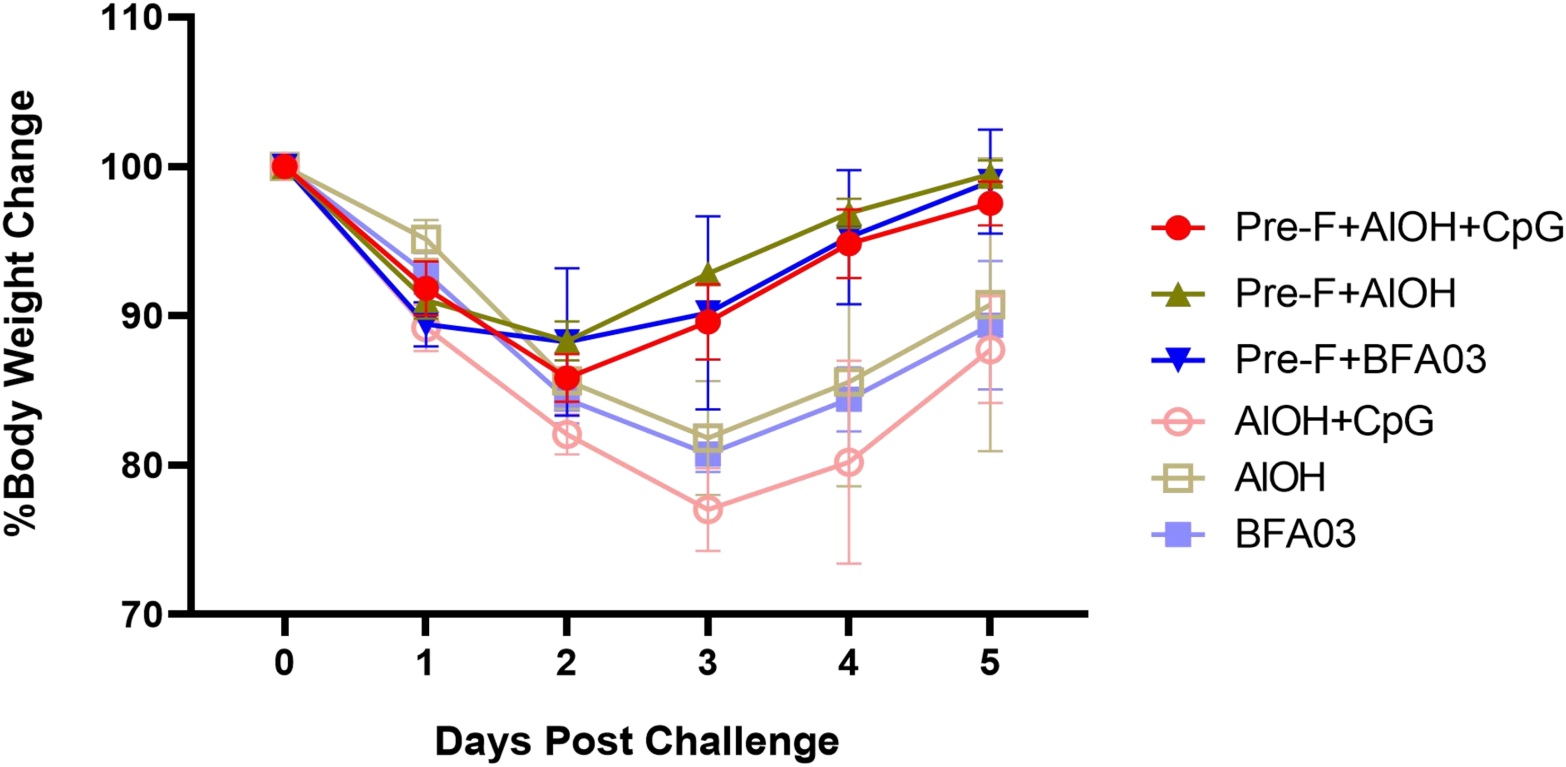
Changes in body weight of mice after virus challenge.

The body weight of the mice was measured every day after challenge (2×10^5^ PFU/mouse). As shown in **Figure 5**, the body weights of mice in the experimental group and the adjuvant group decreased on the first and second days after challenge. However, the body weight of mice in the experimental group began to recover on the third day after challenge, and the body weight of mice in the adjuvant group continued to decrease. On the fifth day after challenge, the body weight of mice in the experimental group recovered to the prechallenge level, but the body weight of mice in the adjuvant group recovered to approximately 90% of the prechallenge level.

## Discussion

Consistent with the results of clinical trials of FI-RSV vaccines, immunization with FI-RSV vaccines induced Th2-biased immune responses and increased lung eosinophils following HRSV challenge in multiple animal models (24). However, the role of eosinophils in mediating ERD has been questioned. Studies have shown that eosinophils cannot directly cause severe immunopathology, and Th2-biased immune responses are necessary to mediate airway hyperresponsiveness and mucus hypersecretion diseases (25). Relevant animal experiments have also confirmed that Th2-biased immune responses caused by FI-RSV are associated with ERD after HRSV infection. Some studies have found that the formalin used for virus inactivation in the FI-RSV vaccine may be related to Th2 bias. After formalin treatment, the protective antigen is destroyed, the antigen produces carbonyl groups, and macrophages target the antigen to scavenger receptors, inducing a Th2-biased immune response and leading to disease enhancement (26). Similar to measles virus (MV), HRSV elicited low-affinity nonprotective antibodies *in vivo* when monkeys were immunized with the formalin-inactivated vaccine against MV, resulting in atypical and severe disease in vaccinated individuals during subsequent MV infection (27). Alternatively, a particular peptide in the G protein (aa 184-196) may contribute to Th2-biased immune responses and eosinophilia (28). The AlOH adjuvant used in many HRSV vaccines induces a Th2-biased immune response (29). In this study, the mice immunized with Pre-F+AlOH+CpG induced higher IgG2a antibody titres than those immunized with IgG1, but the IgG2a/IgG1 ratio of the mice immunized with Pre-F+AlOH was lower than 1. CpG adjuvant can partially counteract a Th2-biased immune response of AlOH adjuvant, and the combination of CpG adjuvant and AlOH adjuvant can induce a more balanced immune response and reduce lung pathological damage in mice. The ratio of IgG2a/IgG1 in the Pre-F+AlOH+CpG group was significantly lower than that in the Pre-F+BFA03 group. Similary, the neutralizing antibody titer in the Pre-F+AlOH+CpG group (1418) was numerically higher than that in the Pre-F+AlOH group (600), and the IgG2a/IgG1 ratio in the CpG-containing adjuvant group also showed a trend toward being higher than that in the AlOH-alone group. Although these differences did not reach statistical significance, the observed directional trends may serve as preliminary biological clues. Nevertheless, they warrant further verification in future studies with larger sample sizes, optimized immunization regimens, or extended sampling time points to determine whether these trends represent genuine biological effects or random fluctuations arising from the limited sample size. Similarly, the lung pathological injury of the Pre-F+ALOH group was the most serious among the experimental groups (score: 2.83). We note that although the mice immunized with Pre-F+BFA03 elicited the highest neutralizing antibody titre (1716), its lung pathology score was slightly higher than that of Pre-F+AlOH+CpG. Neutralizing antibodies are a key of protection, but lung pathology in RSV infection is also influenced by cellular immune responses, inflammatory infiltrates, and immunopathology. This suggests that optimal protection may require a balance between humoral and cellular immunity, rather than max neutralizing antibody titre alone.

BALB/c mice were selected as the animal model because they exhibit higher susceptibility to RSV compared with other mouse strains, possess well-characterized immune response profiles and a mature pathological scoring system, and are widely used in RSV vaccine research. However, RSV replication in the lungs of BALB/c mice is limited and typically requires inoculation with a high viral dose, which cannot fully mimic the deep infection and robust replication observed in the human lower respiratory tract. Therefore, promising candidate adjuvants that yield positive results in BALB/c mice generally require further validation in more relevant models, such as cotton rats or non-human primates, which more closely resemble human RSV infection.

Palivizumab can effectively prevent and treat HRSV-induced infantile bronchitis, indicating that neutralizing antibodies can be prevented without ERD. Weak neutralizing antibodies and immune complexes contribute to ERD (30). Inadequate Toll-like receptor stimulation results in insufficient antibody affinity maturation. TLR agonists (LPS, Poly(I:C), and PolyU) added to FI-RSV vaccines lead to greater affinity maturation and enable vaccine-induced protective responses rather than a disease-enhancing response (31). Original antigenic sin (OAS) may have occurred in children after FI-RSV vaccination, causing the deaths of two HRSV seronegative children and seropositive children who were infected with HRSV before FI-RSV vaccination. HRSV-specific OAS in these children results from natural HRSV infection (32). Therefore, an ideal HRSV vaccine must induce the production of high-titre neutralizing antibodies, and recombinant protein vaccines must rely on an appropriate expression system and powerful new adjuvants. Some studies have shown that the neutralizing antibody titres induced by Pre-F prepared by the *Escherichia coli* expression system and yeast expression system are low, and the recombinant protein vaccines currently in clinical trials are prepared by the CHO cell expression system. In this study, the neutralizing antibody titres induced by AlOH+CpG (1418) and BFA03 (1716) were higher than those induced by the AlOH adjuvant (600). Therefore, to obtain more neutralizing antibodies, HRSV recombinant protein vaccines should not use an AlOH adjuvant.

To measure the effectiveness of a vaccine, we should rely on not only the neutralizing antibody titre but also the comprehensive evaluation of humoral and cellular immunity. In general, the cellular immune response induced by recombinant protein vaccines is lower than that induced by live attenuated vaccines or vector vaccines, but the use of novel adjuvants can significantly improve the strength of the cellular immune response. Aluminium adjuvants induce low cellular immunity, but CpG is a TLR9 agonist that can induce enhanced innate and adaptive immune responses, including cellular immune responses, by stimulating DCs and B cells (33). It can also be seen in the present study that the combination of AlOH+CpG adjuvant induced a higher number of lymphocytes secreting IFN-γ and IL-4 than the AlOH adjuvant group, indicating the ability of CpG adjuvant to enhance cellular immune responses. BFA03 adjuvant induced cellular immune responses of similar magnitude to AlOH+CpG adjuvant.

Vaccine efficacy was also evaluated by changes in body weight and viral load in the lungs of the mice. In this study, the mice in the experimental groups (Pre-F+AlOH+CpG, Pre-F+AlOH, and Pre-F+BFA03) were able to quickly clear the virus in the lung. On the fifth day after challenge, the viral load in the lung of the mice in the adjuvant group increased (the Ct value was approximately 28), while the viral load in the lung of the mice in the experimental group decreased. On the third day of challenge, the mice in the experimental group began to regain weight, but the mice in the adjuvant group continued to lose weight.

In this study, AlOH adjuvant, AlOH+CpG adjuvant, and BFA03 adjuvant (matching GSK AS03 adjuvant) were used to compare the protective effect of Pre-F protein in BALB/c mice. The experimental results showed that the AlOH adjuvant with a Th2-type immune response was not an ideal adjuvant for the HRSV vaccine, and the protective effect of the Pre-F protein combined with the AlOH+CpG or BFA03 adjuvant was similar and better than that of the AlOH adjuvant.

## Data Availability

The original contributions presented in the study are included in the article/supplementary material. Further inquiries can be directed to the corresponding author.
